# PET/CT of the Spleen with Gallium-Oxine-Labeled, Heat-Damaged Red Blood Cells: Clinical Experience and Technical Aspects

**DOI:** 10.3390/diagnostics13030566

**Published:** 2023-02-03

**Authors:** Robert Drescher, Philipp Seifert, Sebastian Gröber, Julia Greiser, Christian Kühnel, Falk Gühne, Martin Freesmeyer

**Affiliations:** Clinic of Nuclear Medicine, Jena University Hospital, Am Klinikum 1, 07747 Jena, Germany

**Keywords:** PET/CT, radiopharmaceuticals, spleen, erythrocytes, oxyquinoline, oxine

## Abstract

Several scintigraphic techniques have been supplemented or replaced by PET/CT methods because of their superior sensitivity, high resolution, and absolute activity quantification capability. The purpose of this project was the development of a PET tracer for splenic imaging, its radiopharmaceutical validation, and its application in selected patients in whom unclear constellations of findings could not be resolved with established imaging methods. Heat-damaged red blood cells (RBCs) were labeled with [^68^Ga]gallium-oxine, which was produced from [^68^Ga]gallium and 8-Hydroxyquinoline (oxine) on an automated synthesizer. Ten patients underwent [^68^Ga]gallium-oxine-RBC-PET/CT for the classification of eleven unclear lesions (3 intra-, 8 extrapancreatic). [^68^Ga]gallium-oxine and [68Ga]gallium-oxine-labeled RBCs could be synthesized reproducibly and reliably. The products met GMP quality standards. The tracer showed high accumulation in splenic tissue. Of the 11 lesions evaluated by PET/CT, 3 were correctly classified as non-splenic, 6 as splenic, 1 as equivocal, and 1 lesion as a splenic hypoplasia. All lesions classified as non-splenic were malignant, and all lesions classified as splenic did not show malignant features during follow-up. PET/CT imaging of the spleen with [^68^Ga]gallium-oxine-labeled, heat-damaged RBCs is feasible and allowed differentiation of splenic from non-splenic tissues, and the diagnosis of splenic anomalies.

## 1. Introduction

Over the years, several scintigraphic techniques in nuclear medicine have been replaced by positron emission tomography/computed tomography (PET/CT) because of its superior sensitivity, high spatial and temporal resolution, exact fusion with morphologic imaging, and the possibility of absolute activity quantification [1,2,3]. The importance of a PET/CT alternative to scintigraphy is emphasized further by the repeated supply shortages of ^99^Mo and thus, ^99m^Tc [4,5].

Differentiation of harmless splenic tissue from malignancies and their metastases can be challenging but is very important, notably in cases in which the suspicious finding is singular and, therefore, decides if the patient is tumor-free. Accessory spleens mimicking metastases or intrapancreatic lesions have been described [6,7,8,9,10]. Particularly regarding the pancreas, avoidance of an unnecessary resection is important since severe complications and pancreatic insufficiencies may arise. Most of the cases described in the literature were solved with spleen scintigraphy. The method relies on the function of the spleen to remove damaged red blood cells (RBCs) from the bloodstream, which are phagocytized by macrophages in the splenic red pulp. Therefore, if damaged RBCs are labeled with radionuclides, their accumulation in splenic tissue can be visualized and quantified.

Indications for splenic imaging include functional assessment, identification of aberrant splenic tissue (hypo-/hypersplenism, asplenia), differentiation of splenic tissue from malignant lesion (which may appear as accessory spleens on conventional imaging and can be located in the pancreas), and further evaluation of developmental anomalies of the spleen [11].

The use of [^68^Ga]gallium-oxine-labeled heat-damaged RBCs for the identification of dystopic splenic tissue and the differentiation of splenic tissue from a neuroendocrine tumor (NET) metastasis on PET/CT was first reported in 2021 [12,13]. These publications also showed a direct comparison of spleen scintigraphy and SPECT images with the results of RBC-PET/CT. We now present a detailed evaluation of the tracer synthesis and biodistribution and the results of clinical applications. 

## 2. Materials and Methods

### 2.1. Synthesis of [^68^Ga]gallium-Oxine 

All syntheses, labeling, and quality control steps were complied with Good Manufacturing Practice (GMP) standards. The automated synthesis of [^68^Ga]gallium-oxine was performed on a Scintomics GRP synthesizer (smartGRP module, SCINTOMICS Molecular, Fürstenfeldbruck, Germany) for labeling of [^68^Ga]gallium peptides (ABX advanced biochemical compounds, Germany). Pharmaceutical grade 8-hydroxyquinoline (oxine) was acquired from Sigma Aldrich (Wien, Austria). The [^68^Ga]gallium chloride radiolabeling solution was eluted with 0.1 M hydrochloric acid from a radionuclide generator (GalliaPharm; Eckert & Ziegler, Berlin, Germany). The syntheses were performed based on a protocol described by Socan et al. [14], using 2 M sodium acetate buffer (pH 5.5) and 150 µg oxine, previously diluted in 150 µL EtOH. The product underwent sterile filtration and was diluted with 10 mL 0.9% saline. Quality control was performed in accordance with relevant monographs of the European Pharmacopeia, e.g., chapters 2.6.1 (sterility) and 2.6.14 (bacterial endotoxins). The monograph on ^68^Ga edotreotide injection was used for guidance regarding the determination methods of radionuclide purity. Special attention was paid to pH (target range 5.9–6.6) and ethanol content determination (target concentration < 7% *v*/*v*) to prevent the RBCs from lysis. Samples were taken for microbiological testing.

### 2.2. Red Blood Cell Labeling 

RBC labeling with [^68^Ga]gallium-oxine followed a standardized labeling procedure, commonly used for ^99m^Tc, as described in guidelines of the European Association of Nuclear Medicine (EANM) and was in accordance with EANM guidelines on current Good Radiopharmacy Practice (cGRPP) in the preparation of radiopharmaceuticals [15]. Venous blood for RBC labeling (8 mL) was taken from the patient with a 10 mL syringe prepared with 2 mL Anticoagulant Citrate Dextrose, Solution A (ACD-A). The subsequent treatment of the cells, up until the preparation of the final injection solution, was performed under a biosafety cabinet (GMP grade A) to ensure an aseptic process environment. The RBCs were separated from the plasma by centrifugation. The supernatant was removed and the sediment was resuspended in 10 mL 0.9% saline, followed by another centrifugation. This washing procedure was repeated until the supernatant was completely colorless. Finally, the RBCs were resuspended in 10 mL of 0.9% saline. 

A total of 300–500 MBq of the [^68^Ga]gallium-oxine solution (6–10 mL) were added to the RBC suspension and agitated gently. To induce heat damaging of the RBCs and to facilitate the RBCs binding to [^68^Ga]Ga-oxine, the mixture was heated to 48 °C for 10 min under gentle agitation. After another centrifugation, supernatant and sediment were separated and each was measured in a dose calibrator. If the supernatant contained more than 5% of the total activity of both supernatant and sediment, indicating the presence of substantial amounts of non-RBC-bound [^68^Ga]gallium-oxine, another washing cycle with 10 mL 0.9% saline, centrifugation, and separation was performed. For injection, the labeled RBC sediment was resuspended in 5 mL 0.9% saline. The mean necessary time interval from blood collection up to the injection of the labeled RBCs was around 2 h.

### 2.3. PET/CT Protocol

The [^68^Ga]gallium-oxine-RBC PET/CT was considered eligible in clinical situations in which differentiation between tumor lesions and splenic tissue had to be made, splenic abnormalities were suspected, and established imaging methods (ultrasound, CT, MR imaging, DOTATOC-PET/CT) were inconclusive. The clinical indications for each patient were confirmed in a multidisciplinary setting, in compliance with the code of ethics of the World Medical Association (Declaration of Helsinki). Patients gave written informed consent.

The American College of Radiology (ACR) and the German Society of Nuclear Medicine (Deutsche Gesellschaft für Nuklearmedizin, DGN) spleen scintigraphy guidelines suggest starting imaging 30 to 120 min and 30 to 120 min, respectively, after injection of ^99m^Tc-labeled, heat-damaged RBCs [16,17]. Due to the shorter half-life of ^68^Ga, PET/CT imaging was performed during 30 min after tracer injection. The protocol was designed to include the upper abdomen. Patients were positioned in the PET/CT scanner (Biograph mCT 40; Siemens Healthineers, Erlangen, Germany) in a supine position. After an unenhanced low-dose CT for anatomical correlation and attenuation correction, an early-dynamic (list mode) PET was acquired continuously for 300 s, beginning with an IV tracer injection. Static PET images (5 min per bed position) were acquired 10 and 25 min after tracer injection. Image reconstruction was performed using the OSEM algorithm with 3D attenuation-weighted ordered subsets expectation maximization at four iterations, 12 subsets with a 5-mm post-reconstruction Gaussian filter, and a 400 × 400 pixel matrix. From the dynamic data, ten subsequent 30 s time intervals (frames) were reconstructed. 

The recommended diagnostic reference value for [^68^Ga]gallium PET tracers is 2.0–2.5 MBq per kg body weight. For the [^68^Ga]gallium-oxine-RBC PET/CT, we used a value of 2.0 MBq/kg body weight with a lower limit of 150 MBq to ensure sufficient image quality for the early-dynamic PET acquisition.

### 2.4. Patient Characteristics

Over two years, ten patients (3 women, 7 men; age: median 62, range 43–77 years) underwent [^68^Ga]gallium-oxine-RBC PET/CT for evaluation of eleven suspicious lesions (Table 1, consecutive order). Clinical indications included differentiation of splenic tissue from carcinoma metastases (6 patients), of pancreatic tumors from intra-pancreatic accessory spleens (3 patients), and detection of functioning splenic remnants in a patient without an obvious spleen on conventional imaging. Five patients were status post splenectomy. All patients gave informed consent. 

### 2.5. PET/CT Evaluation and Quantification

For biodistribution analysis, contouring volumes of interest (VOIs) were drawn over the whole body, organs of visually highest uptake, and bones. Activities in the volumes were determined using the PMOD software package (PMOD Technologies, Zurich, Switzerland). 

Lesions were visually evaluated and quantified by measuring size/volume, mean and maximal standardized uptake values (SUV_mean_, SUV_max_). Volumes of interest (VOIs) for SUV_mean_ and SUV_max_ measurements were also placed in the spleen (if present), liver, kidney, vertebral body L1, back muscle, and abdominal aorta. Lesion-to-muscle ratios were calculated for SUV_max_ and SUV_mean_. Measurements were performed on a syngo.via workstation (MM Oncology module; Siemens Healthineers, Germany).

The lesions were classified by a combination of visual impression and uptake measurements into the categories of splenic tissue, non-splenic tissue, or equivocal. A lesion with a tracer uptake markedly higher than the soft tissue background activity was considered to represent functioning splenic tissue, which would be substantiated by an uptake increase from 10 min to 25 min after tracer injection. Final verification was based on the results of histopathology and follow-up examinations. Lesions were considered benign if no progression occurred within 6 months. 

## 3. Results

### 3.1. PET Tracer Preparation and Quality Control

Following establishment and GMP validation, both [^68^Ga]gallium-oxine and [^68^Ga]gallium-oxine-labeled RBCs could be produced reproducibly and reliably. All quality control results were within specified limits. The pH of [^68^Ga]gallium-oxine solution was between 5.9 to 6.6 and ethanol content was below 7% *v*/*v*. Endotoxin content was ≤ 0.5 IU/mL. Retrospectively, sterility was confirmed for all [^68^Ga]gallium-oxine samples. 

The determination of the radiochemical purity (RCP) of [^68^Ga]gallium-oxine is not possible using standard methods according to Eur. Ph., since [^68^Ga]gallium-oxine is rather unstable and decomposes during radio thin layer chromatography (TLC) and radio high-performance liquid chromatography (HPLC) analysis. Similarly, the labeled RBCs are not suitable to undergo standard radio HPLC. Standard TLC methods (e.g. using silica gel and sodium citrate) for identification of non-bound [^68^Ga]gallium(III) ions lead to the decomposition of the RBCs during the TLC. For this reason, the RCP of the labeled RBCs was determined after centrifugation by measuring the activity in the supernatant and the sediment, respectively. After the required washing steps, the percentage of activity in the sediment in relation to the total activity was defined as the RCP. Mean RCP of the final product was 96.0% (range 95.3–97.2%). 

### 3.2. PET/CT Examinations

Median injected activity was 177 MBq [^68^Ga]gallium-oxine-RBC (range 150–238 MBq). In patient #1, the PET protocol was extended to include static whole-body PET/CTs, acquired 10 and 60 min after tracer injection, respectively. No immediate or delayed complications occurred in any of the investigated patients.

### 3.3. Biodistribution Analysis

**Figure 1:**
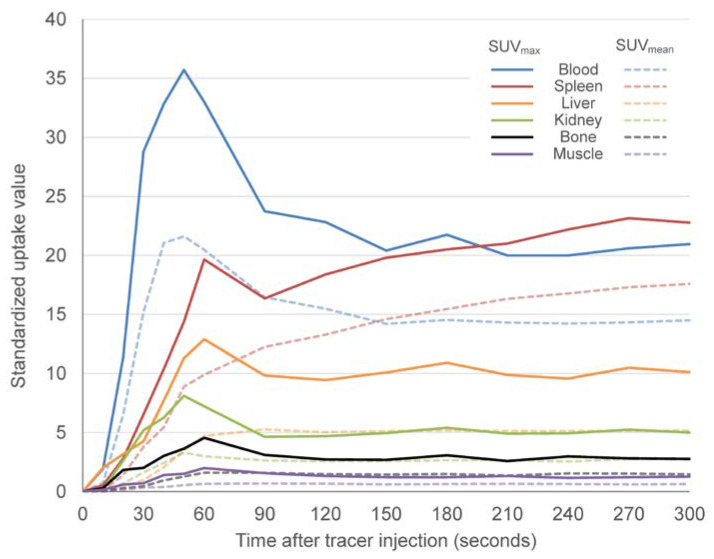
Distribution of [^68^Ga]gallium-oxine in organs/regions on early-dynamic PET/CT.

**Figure 2:**
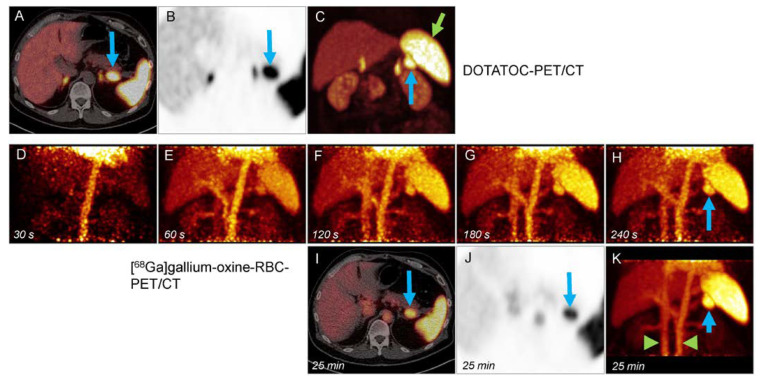
A 62-year-old man (patient #5) with suspected NET in the pancreatic tail. High SSR expression of the ovoid lesion (**A–C**, upper row, blue arrows), nearly equivalent to the spleen (**C**, green arrow). Dynamic (MIP images, **D**–**H**) and late (**I**–**K**) [^68^Ga]gallium-oxine-RBC-PET images show rapid tracer uptake and accumulation in the lesion (beginning between 30 and 60 s after tracer injection (**E**). The mass was classified as splenic tissue. [^68^Ga]gallium-oxine-labeled erythrocytes persist in the blood pool (**K**, aorta, and caval vein, green arrowheads).

**Figure 3:**
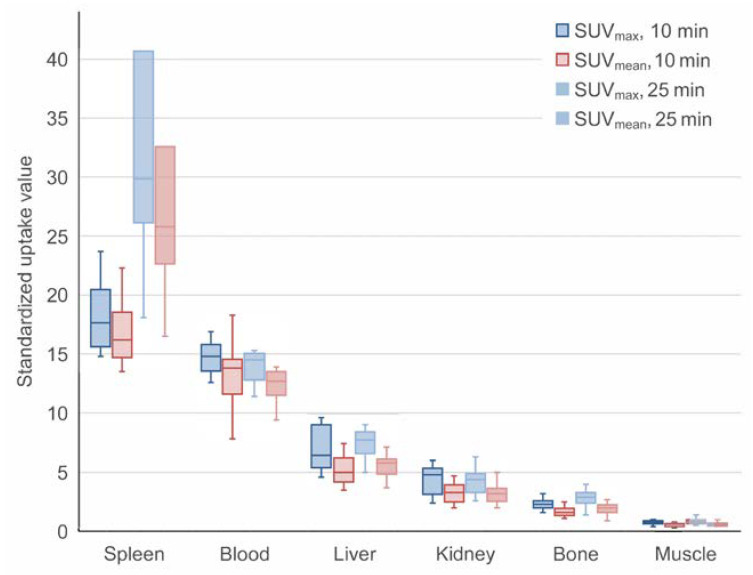
Distribution of [^68^Ga]gallium-oxine labeled RBCs in organs/regions on static PET/CT images. Whiskers represent quartiles.

Four patients who had a native spleen (no. 1, 3, 5, 6) were included in the biodistribution analysis. Early-dynamic PET showed the expected SUV peak in the blood and, less pronounced, in the spleen, liver, kidney, and bone after intravenous injection (Figure 1). Uptake in muscle tissue was constantly low, with only a slight perfusion peak. In the spleen, following a short decrease after the perfusion peak, maximum and mean uptake values increased continually. At the same time, the blood SUV decreased, indicating the removal of marked RBCs from the bloodstream and accumulation in the spleen (Figure 2). In the liver, kidney, and bone, uptake values remained stable from 90 s onwards. Measurements on static images of the upper abdomen 10 and 25 min after tracer injection showed a further increase of the uptake in the spleen, with a further slight decrease in the blood (Figure 2 and Figure 3). Uptake in reference organs did not increase. The blood-to-background ratio remained high. 

Whole-body biodistribution analysis was performed in the first patient examined (patient #1), who underwent whole-body PET acquisitions starting 10 and 60 min after injection of 176 MBq [^68^Ga]gallium-oxine-RBC. At both time points, the highest decay-corrected organ activity proportions were measured in the spleen (Table 2). Between the two time points, the proportion of whole-body activity increased in the spleen, liver, and bone, and remained low in the kidney (only one kidney was present). No activity was seen in the renal collecting system and the urinary bladder. The proportion of activity in the remaining body decreased, from 81% at 10 min to 69% at 60 min after injection. 

### 3.4. Lesion Characteristics

Details of the lesions evaluated in this patient series are shown in Table 1. Three lesions were located in the pancreas, and eight in the left upper abdomen. Patient #9 had lesions in both locations (lesions #9.1, #9.2). The median long axis lesion diameter measured on multi-planar CT was 1.5 cm (range 1.1–4.0 cm), median lesion volume was 1.0 mL (range 0.6–7.0 mL). 

Of the three intrapancreatic lesions, one (lesion #5) showed high tracer uptake typical for splenic tissue, which was confirmed by endoscopic fine-needle aspiration cytology (FNAC). Lesions #3 and #6 showed low tracer uptake (SUV_max_ 1.1 and 3.6 at 25 min) and were classified as non-splenic tissue. Both lesions represented grade 1 NETs. 

**Figure 4:**
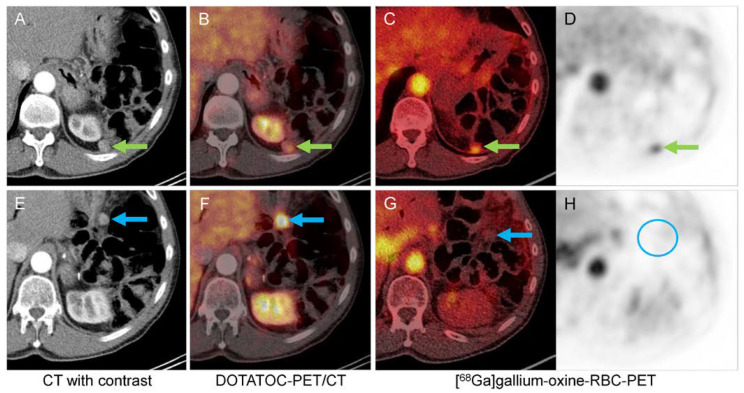
A 68-year-old man (patient #9) with a history of a pancreatic NET and subsequent pancreatic tail resection with splenectomy four years ago. Lesions #9.1 (upper row, adjacent to the diaphragm, green arrows) and #9.2 (lower row, inferior to the stomach, blue arrows) show moderate contrast enhancement (**A**,**E**) and moderate to high SSR expression (**B**,**F**). [^68^Ga]gallium-oxine-RBC-PET/CT showed moderate tracer accumulation in lesion #5.1 (**C**,**D**). No tracer uptake above background activity in lesion #5.2 (**G**,**H**, blue circle), which represented a NET metastasis.

Of the seven lesions in six patients with suspected metastases, five showed moderate-to-high tracer uptake with increasing (lesions #1, #7, #8, and #9.1) or constant (lesion #2) tracer uptake, and were classified as splenic tissue. The second lesion in patient #9 (lesion 9.2) showed very low, constant uptake and was classified as non-splenic tissue (Figure 4). A NET metastasis was confirmed histologically. Lesion #4 showed low uptake (SUVmax at 10 and 25 min: 3.0 and 3.4), but lesion-to-muscle ratios were 5.0 and 4.6, respectively. It was classified as equivocal. After assessing the risks of surgical intervention, the patient underwent imaging follow-up, which did not show evidence of malignant progression.

In patient #10, the heavily calcified structure in the splenic compartment with a diameter of 2.0 cm did not show specific tracer uptake and probably represents a splenic remnant. No functioning splenic tissue was identified in the abdomen and pelvis of the patient. 

No correlation was found between lesion size and tracer uptake. Of the lesions classified as splenic tissue, lesion #8 with high tracer uptake (SUV_max_ 33.7, SUV_mean_ 22.9 at 25 min) was also one of the smallest lesions (volume 0.6 mL). Tracer uptake in the largest lesion (#2) was markedly lower (SUV_max_ 9.3, SUV_mean_ 6.5 at 25 min). 

Tracer uptake in lesions classified as splenic tissue in patients with native spleen (lesions #1 and 5) was not consistently higher or lower compared with these in patients without a native spleen (lesions 2, 7, 8, 9.1). 

**Figure 5:**
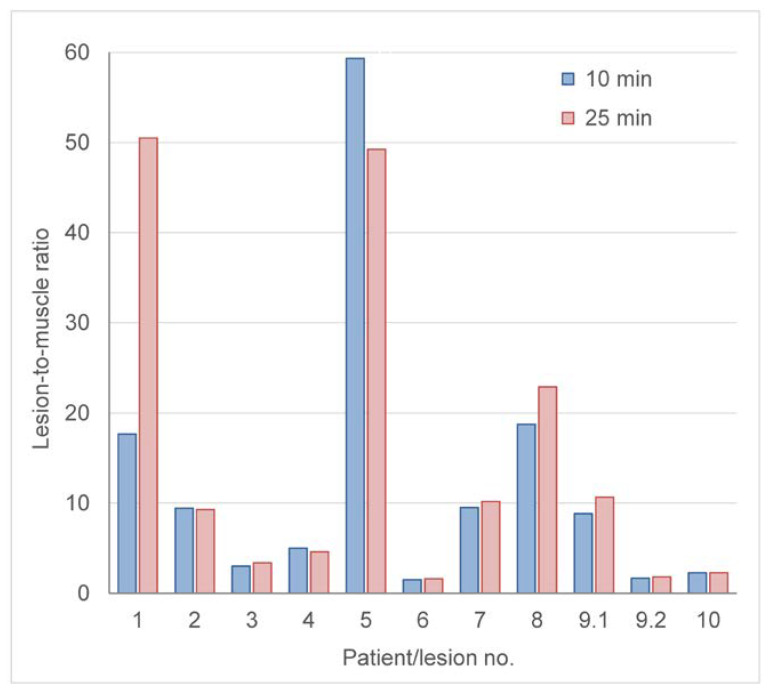
Liver-to-muscle ratio of the suspicious lesions, measured 10 and 25 min after tracer injection. Lesions #3, #6, and #9.2 were classified as non-splenic, lesion #4 as equivocal, and lesion #10 as a non-functioning splenic remnant.

For lesions classified as splenic tissue, the lowest lesion-to-muscle (L/M) ratios were 8.8 (SUV_mean_) and 9.3 (SUV_max_). For lesion #4, which was classified as equivocal on PET/CT, L/M ratios were 5.0 (SUV_max_) and 4.6 (SUV_mean_) (Figure 5).

Histopathologic examinations and follow-up imaging confirmed three malignant and seven non-malignant lesions. On PET/CT, all malignant lesions were classified as non-splenic (100%), whereas six of seven non-malignant lesions were classified as splenic/non-malignant (83%). 

## 4. Discussion

Spleen scintigraphy was developed in the 1960s, using ^198^Au-, ^51^Cr- and ^197^Hg-based tracers, before the advancement of ^99m^Tc [18,19,20,21]. [^68^Ga]Gallium-tracers for positron emission imaging of RBC and platelets, using 8-hydroxyquinolone (“oxine”) as a chelator, were proposed in 1977 [22]. Oxine labeled with ^111^In was the first non-selective labeling agent for white blood cells [23]. In 2017/18, ^18^F-labeled RBCs were successfully evaluated in animal studies [24,25]. However, fluoride-based chemistry requires an on-site cyclotron whereas the production of [^68^Ga]Gallium-oxine became feasible and worthwhile for in-house production in hospital radiopharmaceutical laboratories due to the availability of ^68^Ge/^68^Ga generators and automated synthetization methods [14,26].

Differential diagnoses of upper abdominal masses include benign and malign entities, including accessory/ectopic splenic tissue, non-specifically enlarged lymph nodes, lymph node metastases, extralymphatic soft tissue metastases, manifestations of malignant lymphoma, but also tumors of neural origin (mostly retroperitoneal), and fibroids. All lesions may appear similar on ultrasonography, CT and MR imaging. On PSMA- and DOTATOC-PET/CT, the physiologic tracer uptake of splenic tissue prevents their reliable differentiation from metastases.

In a study evaluating spleen scintigraphy in splenectomized and non-splenectomized patients, it was reported that while specificity is always high, the sensitivity for the detection of accessory spleens is low in non-splenectomized patients [27]. In a series of 10 patients who underwent splenic scintigraphy to further evaluate suspicious findings seen on PET/CT with [^68^Ga]gallium-DOTATOC, only 50% of lesions showed a definite uptake confirming splenic tissue [28]. The negative cases concerned small lesions in patients with the native spleen in situ. The idea to use PET tracers for labeling of blood cells is relatively old but was not introduced into clinical routine for a long time due to the limitations of scanner technology. PET/CT scanners are now widely available, becoming the standard examination for several types of cancer (lung carcinoma, NET, prostate cancer). This, and the expansion of radioligand therapies, promotes an increase in the number of dedicated radiopharmaceutical laboratories. 

### 4.1. PET Tracer Production and Biodistribution

The synthesis of [^68^Ga]gallium-oxine and the radiopharmaceutical labeling of heat-damaged RBCs could be established without major difficulties. Quality control was performed according to GMP regulations and the tracer was considered safe for use on patients when other modalities were exhausted and still inconclusive. Since its introduction, tracer production was reliable without incidents. 

Biodistribution evaluation of [^68^Ga]gallium-oxine-RBC showed that tracer uptake is highest in the spleen, followed by liver and kidney parenchyma. Over time, uptake in the spleen increases while the blood activity decreases, indicating phagocytosis of erythrocytes (Figure 3). Whole-body measurements in one patient showed that after 60 min, 12% of the injected activity was located in the spleen (Table 2). Between 10 min and 60 min, the proportion of activity in the liver more than doubled (4.7% to 9.6%), in part due to non-specific uptake of free [^68^Ga]gallium rather than RBC accumulation alone. Neither biliary nor urinary excretion of activity was evident on PET/CT images, due to the high binding of free ^68^Ga to transferrin in the bloodstream.

### 4.2. Clinical Application

Three groups of patients underwent RBC-PET/CT: (a) patients with a known malignancy and a new lesion suspicious for metastasis, which had been detected with other imaging methods, (b) patients with a newly diagnosed pancreatic mass which could, based on MR imaging, be a malignant tumor or an accessory spleen, and (c) one patient with the clinical diagnosis of asplenia. In nine out of ten patients, the clinical challenge was the differentiation of splenic tissue from other tissue structures in the left upper abdomen, where most accessory or residual spleens are detected, or in the pancreas, where accessory spleens may appear as malignant tumors on other imaging methods. Most commonly, the dilemma arose on surveillance examinations of NET patients, because highly differentiated NETs as well as splenic tissue express somatostatin receptors (SSR) and, therefore, are positive on DOTATOC-PET/CT. In six cases, the lesions in question had a typical appearance of splenic tissue on RBC-PET/CT, and unnecessary surgery was avoided. In one pancreatic lesion, additional FNAC, which was a novel method at the time of this study, was performed due to the clinical significance of the diagnosis; however, splenic tissue was confirmed. In all cases in which the lesions were classified as non-splenic on RBC-PET/CT, they represented malignancies. 

The initial classification of lesions as splenic or non-splenic tissue was performed visually, based on the experience with spleen scintigraphy. To objectivize the results, we looked for a numerical threshold (SUV_mean_, SUV_max_, ratios). However, in our patient cohort, SUV values in lesions classified as splenic tissue were highly variable (Table 1). To calculate a lesion-to-background ratio, the uptake in muscle was the preferred reference value because it was consistently low in all patients (Figure 1, purple lines). A lesion-to-muscle ratio of 5:1 may be helpful to support the decision between the two tissue types. The single lesion in this series which was seen as equivocal (lesion #4) appeared benign on follow-up imaging. 

In one patient with suspected hyposplenism, the RBC-PET/CT was performed to identify dystopic splenic tissue, because a functioning spleen would have been important because of the planned immunosuppressive treatment of long-term rheumatoid arthritis. Hyposplenism is often associated with sickle cell anemia and celiac disease, but congenital forms have been described [29,30]. In this patient, no functioning splenic tissue was found. 

In this evaluation of [^68^Ga]gallium-oxine-RBC-PET/CT, no correlation between uptake and lesion size was seen, probably due to the superior spatial resolution of PET/CT, which avoids volume averaging. 

## 5. Conclusions

In conclusion, it could be shown that labeling of heat-damaged RBCs with [^68^Ga]gallium-oxine is feasible, reliable, and can be performed under GMP conditions in a clinical radiopharmaceutical laboratory. PET/CT imaging with the radiotracer has been successfully performed in patients to identify splenic tissue. No complications occurred. The method, while utilizing the superior sensitivity as well as spatial and temporal resolution of PET/CT, may overcome the limitations of standard [^99m^Tc]-pertechnetate RBC scintigraphy, and may serve as a backup method during periods of ^99m^Tc shortage.

## Figures and Tables

**Table 1:** Characteristics of patients and lesions.

Patient		Suspicious Lesion(s)												Consequence
Lesion No. (#)	Primary Diagnosis	Localization	Differential Diagnosis	CT			PET							
				Shape	Diameter	Volume	SUV_max_		SUV_mean_		L/M Ratio	L/M Ratio	Lesion Classification	
					(cm)	(mL)	10 min	25 min	10 min	25 min	10 min	25 min		
1	ccRCC	left upper abdomen	metastasis	ovoid	1.1	2.0	17.7	29.1	5.3	20.2	17.7	50.5	splenic tissue	structured follow up
2	NET	left upper abdomen	metastasis	lobulated	4.0	7.0	9.2	9.3	6.6	6.5	9.4	9.3	splenic tissue	structured follow up
3	pancreatic mass	pancreatic tail	pancreatic NET	ovoid	1.5	1.0	2.6	3.6	1.8	2.7	3.0	3.4	non-splenic tissue	FNAC, SSA therapy
4	NET	left upper abdomen	metastasis	flat	2.8	1.2	3.0	3.4	2.0	2.3	5.0	4.6	equivocal	structured follow up
5	pancreatic mass	pancreatic tail	pancreatic NET	ovoid	3.1	4.7	25.6	26.8	17.8	19.7	59.3	49.3	splenic tissue	FNAC, follow up
6	pancreatic mass	pancreatic tail	pancreatic NET	ovoid	1.5	1.3	1.0	1.1	0.9	0.8	1.5	1.6	non-splenic tissue	resection
7	NET	left upper abdomen	metastasis	flat	1.3	0.6	5.7	7.6	3.8	5.1	9.5	10.2	splenic tissue	structured follow up
8	NET	left upper abdomen	metastasis	ovoid	1.2	0.6	22.3	33.7	15.0	22.9	18.8	22.9	splenic tissue	structured follow up
9.1	NET	left upper abdomen	metastasis	ovoid	1.5	0.7	8.0	9.6	5.3	6.4	8.8	10.7	splenic tissue	structured follow up
9.2	NET	left upper abdomen	metastasis	ovoid	1.1	0.7	1.6	1.6	1.0	1.1	1.7	1.8	non-splenic tissue	resection
10	no spleen	left upper abdomen	asplenia	ovoid	2.0	0.8	2.3	2.4	1.6	1.6	2.3	2.3	splenic hypoplasia	no further imaging

CT, computed tomography; PET, positron emission tomography; SUV, standardized uptake value; L/M ratio, lesion-to-muscle ratio (SUVmean); ccRCC, clear-cell renal cell carcinoma; NET, neuroendocrine tumor; FNAC, fine-needle aspiration cytology; SSA, somatostatin analogue.

**Table 2:** Activity distribution on whole-body PET/CT of patient #1.

Body Structure	Activity, MBq ^1^ (Proportion of Total, %)	
	10 min	60 min
whole body	159.0 (100%)	91.0 (100%)
spleen	12.2 (7.7%)	11.3 (12.4%)
liver	7.5 (4.7%)	8.7 (9.6%)
kidney	0.9 (0.6%)	0.5 (0.5%)
bone	10.1 (6.4%)	7.9 (8.7%)
remaining tissue	128.3 (80.7%)	62.6 (68.8%)

^1^ decay corrected.

## Data Availability

Data sharing is not applicable to this article.
